# Supplementary material for: The mycotoxin Beauvericin is an uncompetitive inhibitor of Cathepsin B
Source: Protein Sci. 2025 May 24;34(6):e70173. doi: 10.1002/pro.70173 (PMC12102733; doi:10.1002/pro.70173)
Supplement: Supplementary file 1 — Data S1. Supporting Information. [file PRO-34-e70173-s001.docx]

**Supplementary Materials for**

# **The mycotoxin Beauvericin is an uncompetitive inhibitor of Cathepsin B**

Xiaoli Yang^1*^, Pablo Cea-Medina^2*^, Mohanraj Gopalswamy^2^, Aparna Vaidya^1^, Sonja Schavier^1^, Shixin Oltzen^3^, Sofie Moßner^3^, Anfei Huang^4^, Jing Qi^5^, Johanna Maria Hölken^6^, Nicole Teusch^6^ Doreen M. Floss^3^, Markus Uhrberg^5^, Holger Gohlke^2,7†^, Stefanie Scheu^1,8†^

^1^Institute of Medical Microbiology and Hospital Hygiene, Medical Faculty and University Hospital Düsseldorf, Heinrich Heine University Düsseldorf, D-40225 Düsseldorf, Germany.

^2^Institute for Pharmaceutical and Medicinal Chemistry, Heinrich Heine University Düsseldorf, D-40225 Düsseldorf, Germany.

^3^Institute of Biochemistry and Molecular Biology II, Medical Faculty and University Hospital Düsseldorf, Heinrich Heine University Düsseldorf, D-40225 Düsseldorf, Germany.

^4^Würzburg Institute of Systems Immunology, University of Würzburg, D-97078 Würzburg, Germany.

^5^Institute for Transplantation Diagnostics and Cell Therapeutics, Medical Faculty and University Hospital Düsseldorf, Heinrich Heine University Düsseldorf, D-40225 Düsseldorf, Germany.

^6^Institute of Pharmaceutical Biology and Biotechnology, Heinrich Heine University Düsseldorf, Universitätsstraße 1, 40225 Düsseldorf, Germany.

^7^Institute of Bio- and Geosciences (IBG-4: Bioinformatics), Forschungszentrum Jülich, D-52425 Jülich, Germany.

^8^Institute of Immunology, Rostock University Medical Center, D-18057 Rostock, Germany.

^*^ These authors contributed equally

^†^ These authors share senior authorship

Correspondence

Corresponding author 1: Stefanie Scheu, Institute of Immunology, Rostock University Medical Center, Schillingallee 70, D-18057 Rostock, Germany, +49 381 494 5870, stefanie.scheu@med.uni-rostock.de

Corresponding author 2: Holger Gohlke, Institute for Pharmaceutical and Medicinal Chemistry, Heinrich Heine University Düsseldorf, Universitätsstr. 1, D-40225 Düsseldorf, Germany, +49 211 81 13662, gohlke@uni-duesseldorf.de

**S1. Experiment section**

**S1.1 SDS-PAGE for CTSB**

10 μg of activated or non-activated CTSB were incubated in SDS loading buffer (400 mM DTT, 200 mM Tris/HCl pH 6.8, SDS 8%, Bromophenol blue 0.4%, glycerol 40%) for 5 minutes at 95 °C. The samples were loaded into a PROTEAN TGX Precast polyacrylamide gel (BioRad). Electrophoresis was performed first at 200 V for approximately 10 minutes (until the sample left the concentrator) and then at 110 V. The gel was stained by submerging it in a solution containing Coomassie Brillant Blue R-250 and then destained in a solution of 50% methanol and 10% acetic acid.

**S2. Figures and Legends**

1. SDS-PAGE of inactive and active CTSB (Supplementary Figure 1)


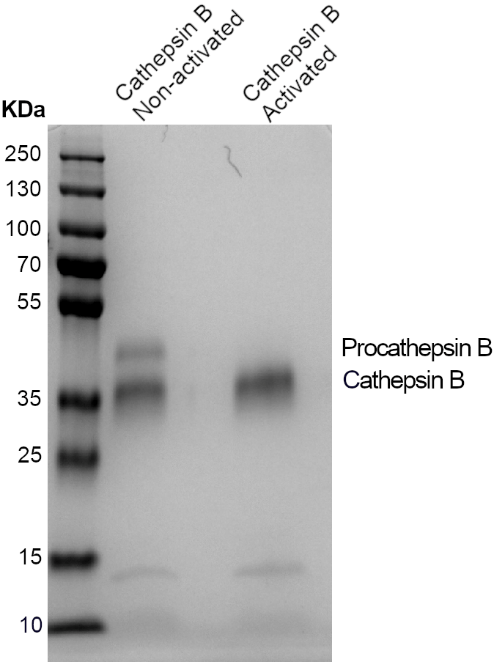


**Supplementary Figure 1**. SDS-PAGE of inactive (left lane) and active CTSB (right lane). The activation process fully converts Procathepsin B into its cleaved form. No other bands in the size range of CTSB are observed.
